# From ilmenite to TiO_2_ nanostructures: integrated processing routes, phase control, and photocatalytic applications

**DOI:** 10.1186/s11671-026-04554-1

**Published:** 2026-04-20

**Authors:** Mohammad Sadegh Moradi, Maryam Yaldagard, Amin Alamdari, Siamak Salehian

**Affiliations:** Department of Chemical Engineering, Faculty of Engineering, Urmia University, Urmia, Iran

**Keywords:** Titanium dioxide, Photocatalyst, Ilmenite, Rutile, Anatase

## Abstract

In recent years, processing naturally occurring materials, such as ilmenite, to develop titanium dioxide nanostructures has emerged as an economic, renewable, and eco-friendly approach. This review article focuses on various processing routes of ilmenite, namely, pre-treatment, extractive processing (sulfate and chloride methods), and the synthesis of the resulting titanium dioxide nanostructures. The resulting architectures, including nanofibers, nanotubes, nanowires, and nanoparticles, demonstrate significant potential in photocatalytic applications, particularly under visible light irradiation. While there have been encouraging outcomes at laboratory scales, significant challenges remain, such as the diversity in chemical compositions of ilmenite deposits and the comparatively high energy consumption associated with conventional processing routes. Addressing these challenges requires the development of energy-efficient processing routes (100–120 °C) as an integral component of industrial scalability. Overall, utilizing ilmenite as a precursor for nanostructure synthesis holds immense potential for promoting green technologies in environmental, energy, and healthcare sectors.

**Supplementary Information:**

The online version contains supplementary material available at 10.1186/s11671-026-04554-1.

## Introduction

Titanium dioxide (TiO_2_) has long been of great scientific and industrial importance due to high chemical stability, low toxicity, and relatively low production cost. In nanostructure configurations, like in nanoparticles, nanotubes, nanowires, and nanosheets, TiO_2_ exhibits high surface area and good photocatalytic activity, and it also holds central position in renewable energy, environmental purification, and emerging technologies [1–3]. It would indeed be apt here to say that TiO_2_ has been comprehensively surveyed for solar-driven hydrogen production due to photocatalytic water split and removal of undesirable organic polluters like dyes, pharmaceuticals, and pesticides [4–7]. It also shows antimicrobial characteristics and extends to usability in healthcare and in biomedical applications [8].

The photocatalytic ability of TiO_2_ emerges as an outcome of UV-light absorbency, pushing electrons from the valence to the conduction band, forming hole–electron pairs in order to initiate reactive oxygen species [9, 10]. The latter catalyse oxidation and reduction reaction pathways to support pollutant removal and H_2_ production. However, as a consequence of its high band gap (3.2 eV for anatase, 3.0 eV for rutile) absorption happens to a great degree in the UV part, while solar radiation comprises basically visible radiation [10]. To reduce this constraint, an enormous series of strategies have been examined, i.e., doping by noble metals (Pt, Ag, Au), transition metals (Fe, Ni, Cu, Co), hybridization by metal and non-metallic oxides, and inclusion in carbon-containing materials such as in graphite and graphene [11–15]. Aside from this photocatalytic field, TiO_2_ also features in dye-sensitized solar cells and Li-ion batteries due to its stable structure, while still, rutile TiO_2_ remains an essential white pigment in coatings, in inks, plastics, and paint due to its brilliance and stability [16–19].

Preparation of TiO_2_ nanostructures has been achieved through various routes, i.e., sol–gel, hydrothermal, solvothermal, and deposition and also through electrochemical routes [20, 21], commonly utilizing precursors of titanium isopropoxide, titanium butoxide, titanium tetrachloride, and titanium oxysulfate [13]. However, large-scale exploitation of naturally occurring minerals, like ilmenite, leucoxene, and rutile sands, continues to remain small scale. Among these, ilmenite occurs to be most abundant but inevitably contains impurities in the form of iron oxides, silica, and manganese oxides with potential to compromise photocatalytic functionality [22, 23]. Effective synthesis and extractive schemes from ilmenite are therefore essential to realizing cost-effective and sustainable TiO_2_-based technologies. This review provides an in-depth analysis of ilmenite-based TiO_2_ nanostructures, strong emphasis being placed on synthesis schemes, morphology control, and functional attributes, as a route to industrial translation.

## Global reserves of titanium-bearing minerals

Ilmenite appears as a dark-colored iron–titanium dioxide mineral and also shows an anisometric, hexagonal crystal structure. It also shows 5–6 Mohs hardness and commonly appears in association with other sulfide and oxide phases, such as magnetite, hematite, rutile, and pyrrhotite. As per United States Geological Survey (USGS) statistics, in 2019, roughly 90% of titanium minerals consumed in production were utilized in terms of pigments of TiO_2_, whereas 10% utilized in welding consumables and minor industrial applications [24, 25]. The world’s reserves of ilmenite, in 2019, accounted to be over 880 million tons.

**Fig. 1:**
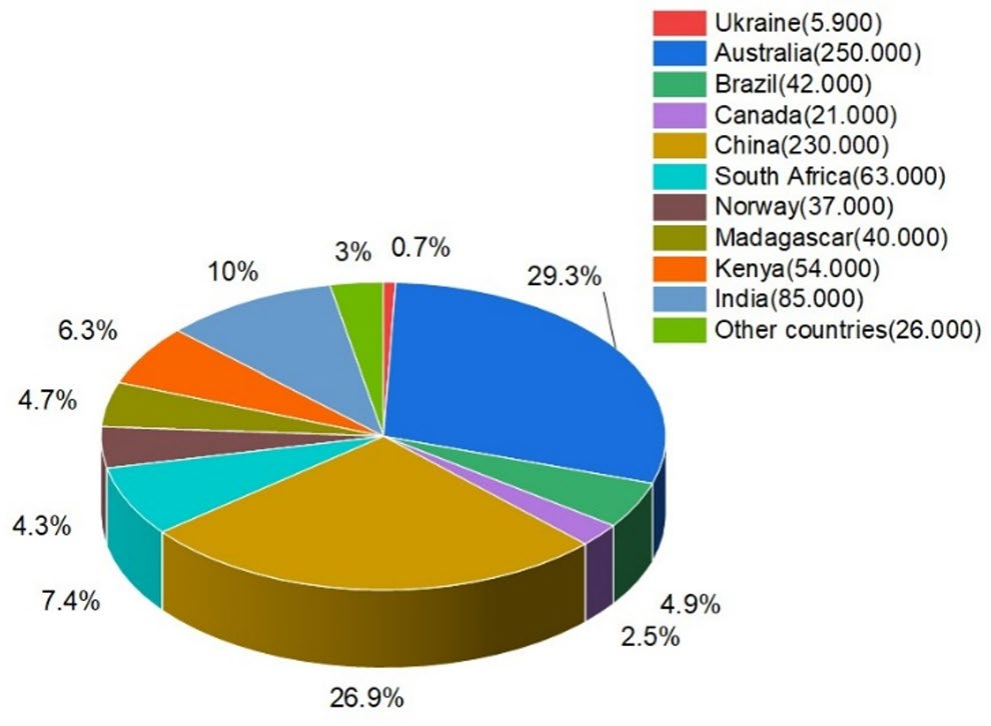
Global reserves of ilmenite mineral [29]

In Indonesia, there are extensive deposits of ilmenite throughout South and Central Kalimantan and in Bangka–Belitung Islands. The Bangka–Belitung ilmenite deposits occur in association most typically with cassiterite (SnO_2_) and have an average 51.21% TiO_2_ content [26]. The Kalimantan ilmenite, in contrast, occurs more typically in association with chromium-bearing phase minerals [27]. X-ray fluorescence (XRF) tests on samples taken throughout these regions demonstrated TiO_2_ as the dominant oxide at an average value of approximately 49.87%, in secondary association with Fe_2_O_3_, Cr_2_O_3_, Al_2_O_3_, and SiO_2_ [28]. The global location of deposits of ilmenite can be observed in Fig. 1 [29], indicating both geographical dispersal and potential access to this critical mineral resource.

**Fig. 2:**
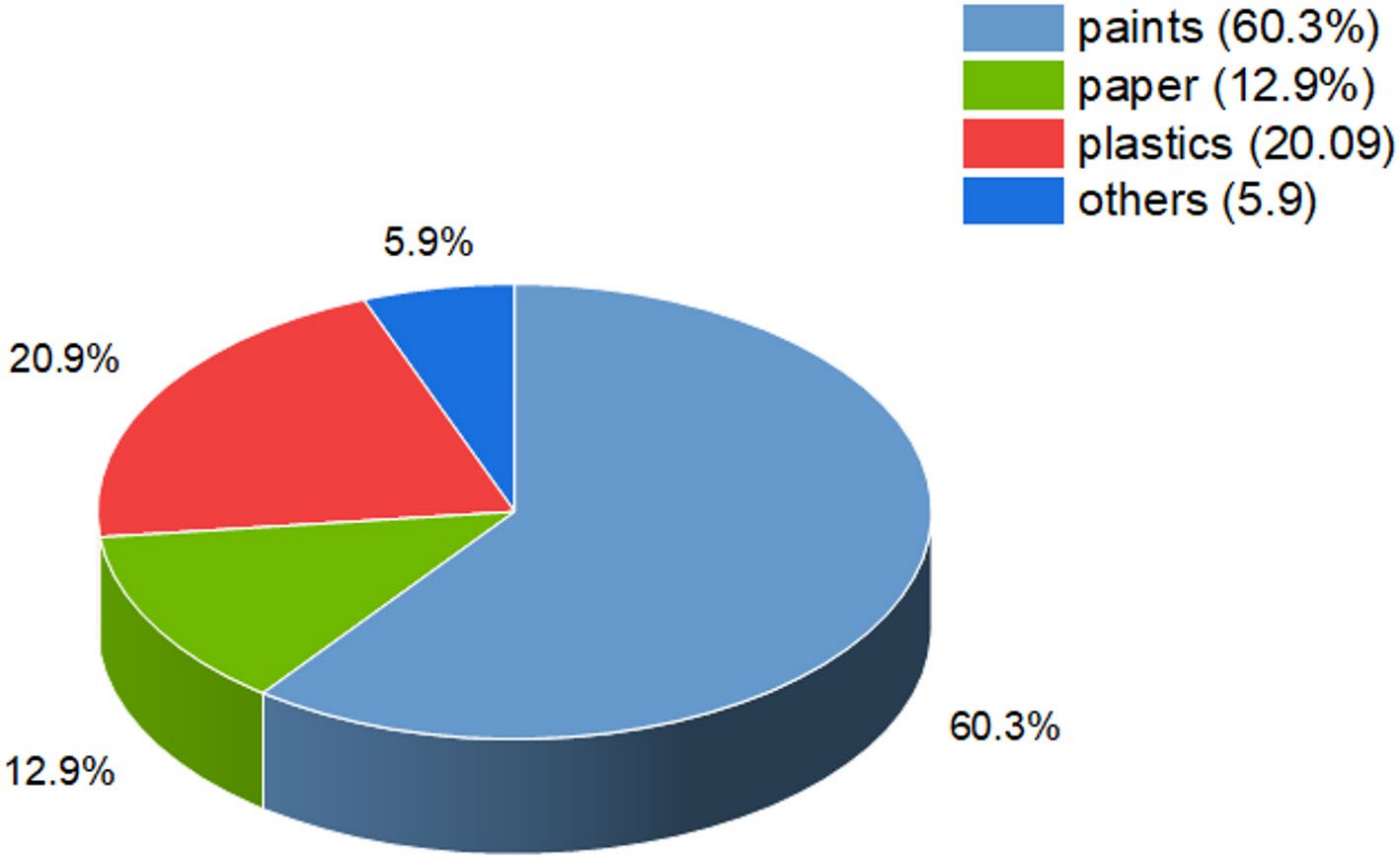
Global titanium dioxide market by application [30]

The utilization of ilmenite as one of the precursors to develop TiO_2_ nanostructures offers an economic and strategic means to create economic value while keeping production costs to a minimum. The 2022 world market of TiO_2_ was dominated mainly over paints (60.3%), plastics (20.9%), paper (12.9%), and other applications, including inks, coloring in foods, and cosmetics (5.9%). The extensive natural reserves of ilmenite, in combination with advances in scalable and efficient synthesis methods, provides an ecologically and economically feasible means to commercialize this ore, identifying it as playing a central role in today's technological and industrial applications. The world market overview of titanium dioxide by end-use is described in Fig. 2 [30], giving an intuitive representation of sectoral segmentation.

Strategically utilizing ilmenite can offer excellent economic and technological benefits, such as less reliance on scarcer and more expensive titanium sources, less production costs, better industrial competitiveness, and broader applications of TiO_2_ nanostructures in catalysis, energy storage, and cleaning of the environment, etc. This study focuses on an exploration of synthesizing TiO_2_ nanostructures from ilmenite by different methodologies, and provides an exhaustive analysis of their structure, morphology, and functional properties, as well as potential performances in different applications. By systematically correlating synthesis parameters and material properties, this study tries to offer an optimal framework for utilizing ilmenite-based TiO_2_ nanostructures in industrial and technological applications.

## Ilmenite processing

### Pre-treatment process

The titanium has to undergo an important pre-treatment stage prior to extracting it from ilmenite to reduce impurities and have consistency in terms of particle size to ensure elevated extraction efficiency. The initial process typically involves washing and drying of the ore, and this is presently the most utilized methodology to remove surface contaminants [31]. Beyond these typical techniques, advanced techniques, i.e., mechanical activation [32], oxidative/reductive processing [33], and hydrothermal pre-treatment, have been employed not only to adjust particle size but also to elevate titanium content in the ore. Among these, mechanical activation has emerged as prominent, as it can reduce particle size efficiently and simultaneously boost leachability without inputting excessive energy and without creating an extensive-scale adverse ecological impact [34]. These features allow mechanical activation to be an efficient and eco-friendly option as regards ilmenite preparation.

### Mechanical activation

Mechanical and thermal activation are two most widely used pre-treatment methods to treat ores in order to elevate mineral solubility. Mechanical activation enhances mineral solubility through particle size reduction and the induction of mechanochemical responses, which in turn modify the material's composition and structure. It includes both dominant and subordinate activities: principal effects include an increase in material's internal and surface energy, specific surface area, and solid cohesion energy reduction, which in combination increase reactivity in the mineral. The secondary effects, such as agglomeration, adsorption, and recrystallization, emerge during or after milling [35].

Ball milling, relying on steel balls colliding with particles of ore, disintegrates the particles and reveals new reactive surfaces. The results of mechanical activation involve reduction in size of particles, increased degree of amorphization, and transformation of the crystal structure, all of which significantly contribute to improvement in the efficiency of extraction. In addition, different parameters of mechanical activation have been examined and utilized in order to maximize extraction yields, showing significance in terms of optimization of process condition [36]. A comparative overview of the advantages, disadvantages, and overall applicability of key pre-treatment methods, including ball milling, oxidation/reduction, and hydrothermal processing, is summarized in Table 1. This table highlights that while ball milling significantly enhances reactivity and surface area, it may introduce contamination and requires careful control of particle uniformity. In contrast, hydrothermal methods offer high titanium recovery but pose environmental challenges due to the use of concentrated acids and emission of toxic gas.

**Table 1:** Advantages and disadvantages of the pre-treatment process for ilmenite mineral

Pre-treatment method	Advantages	Disadvantages	Overall assessment	References
Ball milling	1. Increased specific surface area 2. Crystal structure modification 3. Enhanced mineral reactivity	1. Sample contamination from milling equipment 2. Irregular particle shapes, reduced uniformity	Significantly improves reactivity, but careful attention is required to minimize contamination and maintain particle uniformity	[34, 35, 41]
Oxidation/reduction	1. Formation of microcracks 2. Phase transformations facilitating extraction	1. High energy consumption 2. CO_2_ emissions	An effective method, yet energy-intensive with environmental concerns, requiring careful management of energy use and gas emissions	[33]
Hydrothermal	1. Impurity reduction 2. High titanium recovery (80–85%)	1. Use of concentrated acids 2. Emission of toxic gases (H_2_S, SO_2_, SO_3_)	Highly efficient with excellent recovery, but poses significant environmental challenges and requires precise process control	[31]

Several parameters during ball milling have been found to considerably influence reduction in size of particles and subsequent extraction efficiency of ilmenite. Among these parameters, milling time has been found to be one such critical variable. An increase in milling time to 30 min has been reported to considerably diminish size of particles from an initial size of 140 μm to below 2 μm, and also induce lattice strain to cause broadened and diminished X-ray diffraction peaks. These types of structure modifications enhance reactivity and solubility of the mineral. While there may be a possibility of increased milling time resulting in an agglomeration of particles, preventing size diminution, increased kinetic energy imposed on particles results in enhanced extraction efficiency [37].

Speed of milling also plays an overriding role in establishing particle size development. Medium to high rates of rotation accelerate particle comminution, fast-tracking minimum size in short periods of time. Ball size selection is also critical, as it must find a compromise between frequency of collision and impact energy; medium ball sizes have been reported to offer optimal reduction in size of particles. What's more, wet milling presents certain advantages over and beyond dry milling. The fact that there has to be a fluid medium ensures rapid size reduction of particles, avoids agglomeration, and offers increased specificity in surface area. The fact that water also offers surfactant properties ensures superior production of micro-cracks on surface corners of particles, and an enhancement in titanium leachability. In laboratory cases, titanium leachability in wet process under 90 °C sulfuric acid yielded 72% recovery, compared to 54% in dry milling [38–40].

The ratio of ball/powder also happens to be another critical parameter involved in this process. The increase in this ratio leads to an increase in ball/particle collision numbers and, therefore, raises size reduction. As can be witnessed, an increase in this ratio from 1:6 to 1:1 has been cited to increase considerably the extractive efficiency and reduce particles' size. Separately, optimization of milling time and speed, selection of milling medium, and ball/powder ratio have been significant means of boosting efficiency in ilmenite processing, improving yields, and reducing operational costs [41].

### Ilmenite extraction

Ilmenite is recognized as one of the most cost-effective and abundant sources for titanium dioxide (TiO_2_) production, particularly due to its lower price compared to rutile and other titanium-rich minerals. Industrially, TiO_2_ extraction from ilmenite is primarily achieved through two main pathways: the sulfate process and the chloride process, accounting for approximately 40% and 60% of global TiO_2_ production, respectively [22].

The sulfate process holds strategic importance for processing low- to medium-grade ilmenite (TiO_2_ content 40–65%), owing to the global abundance of such ores. However, its environmental liabilities must be critically addressed, particularly the generation of large volumes of FeSO₄•7H_2_O waste and acidic effluent, which pose significant disposal challenges [22, 46]. Process parameters, including acid concentration (8–12 M H_2_SO₄), temperature (80–120 °C), and solid-to-liquid ratio, should be optimized not only to maximize extraction efficiency but also to minimize iron co-dissolution and reduce downstream purification requirements [31, 42, 44]. A key insight is that mechanical activation of ilmenite prior to sulfuric acid leaching can lower acid consumption and reduce the required operating temperature, thereby improving both the economic and environmental profiles of the process [31, 36, 37].

The chloride process is recognized for its superiority in producing high-purity, photograde TiO_2_, predominantly in the rutile phase. However, its constraints must be critically examined, including its dependence on high-grade feedstock such as rutile or upgraded titanium slag, as well as the handling of hazardous reagents like Cl_2_ and TiCl₄ [22, 23]. Although this route generates less waste compared to the sulfate process, its high capital and operational costs, coupled with stringent safety requirements, limit its applicability in regions where low-grade ilmenite is abundant. This comparative analysis underscores that the selection between sulfate and chloride processes is influenced not only by technical considerations but also by geoeconomic and regulatory factors.

Both routes are based on hydrometallurgical principles, which offer notable advantages over pyrometallurgical or electrometallurgical methods, including higher product purity and reduced operational costs. The key distinction between the two processes lies in the feedstock requirements and the underlying chemical reactions. The sulfate process allows the use of low-grade ilmenite, which can be dissolved in concentrated sulfuric acid. Following the removal of impurities, the solution undergoes hydrolysis to yield hydrated TiO_2_. In contrast, the chloride process necessitates high-value feedstock such as rutile or titanium-rich slag, which are chlorinated in the presence of coke to produce TiCl₄. This intermediate is subsequently converted into TiO_2_ through oxidation or reaction with AlCl_3_.

While the TiO_2_ produced via the sulfate route generally has lower crystallinity and purity compared to the chloride-derived product, the widespread availability of ilmenite, roughly ten times that of rutile, makes the sulfate process economically attractive and operationally feasible [22]. Extensive research has demonstrated that various parameters significantly influence extraction efficiency in the sulfate route. These include sulfuric acid concentration [42], particle size [36, 43], leaching time and temperature [31], and the acid-to-ore ratio [44]. Careful optimization of these factors is essential to maximize yield and process efficiency.

Ball milling is highlighted not merely as a pre-treatment step, but as a mechanochemical activation process that induces structural disorder, increases specific surface area, and enhances leaching kinetics without requiring excessive acid or elevated temperature [34, 36, 37]. For example, Li et al. [37] reported that 30 min of wet milling reduced ilmenite particle size from approximately 140 µm to below 2 µm, subsequently increasing titanium extraction in sulfuric acid from 54 to 72% at 90 °C. Such energy-efficient intensification strategies are considered essential for scaling ilmenite processing in a sustainable manner.

Reductive leaching, employing metallic iron (Fe⁰) in hydrochloric acid, selectively dissolves iron while minimizing titanium loss, yielding a TiO_2_-rich residue that serves as an excellent precursor for pure or doped TiO_2_ nanostructures [49, 53, 71]. For instance, El-Hazek et al*.* [49] achieved near-complete dissolution of Fe and Ti from ilmenite using 12 M HCl with Fe⁰ addition, producing a TiO_2_ concentrate amenable to hydrothermal nanotube growth. Similarly, acid mixtures (e.g., HF + H_2_SO₄) can enhance titanium extraction from refractory ores but may introduce fluoride ions that affect subsequent hydrolysis and crystallization steps [56].

Post-leaching treatments, such as hydrolysis, calcination, and solvent extraction, also play a decisive role in determining the final TiO_2_ phase. For example, hydrolysis in alcoholic media (e.g., 2-propanol) tends to favor the anatase phase at lower temperatures (≤ 550 °C), whereas aqueous hydrolysis followed by high-temperature calcination promotes rutile formation [73]. This ability to tailor phase composition during the extraction and post-treatment stages is crucial for photocatalytic applications, in which anatase is generally preferred due to its higher photocatalytic activity [10, 45].

Emerging approaches such as microwave-assisted leaching [56], solvent extraction with D2EHPA for purifying leachates [57], and the use of ammonium salts (e.g., NH₄Cl) as lixiviants in aeration leach systems [72] are discussed. These methods offer potential improvements in selectivity, kinetics, and waste reduction, but require further development for industrial adoption.

It is argued that extraction should not be isolated from downstream applications. For photocatalytic TiO_2_, minor impurities such as Fe^3^⁺ or Mn^2^⁺ can act as dopants that enhance visible-light absorption, suggesting that controlled impurity retention during leaching may be beneficial rather than detrimental [83, 85]. This perspective contrasts with traditional pigment production, where ultra-high purity remains paramount.

Rather than treating FeSO₄ as waste, it could be valorized into iron pigments, fertilizers, or energy storage materials. Similarly, silica and other impurities removed during leaching could find use in construction materials. We reference studies that achieve > 99% Fe extraction with TiO_2_ residues exceeding 90% purity, demonstrating the feasibility of near-zero-waste processing [71, 72].

A critical tension is noted: many so-called "green" synthesis routes for TiO_2_ nanomaterials still depend on energy-intensive, waste-generating extraction methods. Truly sustainable TiO_2_ production from ilmenite would require low-temperature leaching (< 120 °C), the use of renewable or recyclable lixiviants, and full by-product utilization. The promising role of organic acids (e.g., H_3_PO₄) and bio-derived reagents as potential alternatives to mineral acids is highlighted [60], although their scalability remains unproven.

It is argued that extraction should not be isolated from downstream applications. For photocatalytic TiO_2_, minor impurities such as Fe^3^⁺ or Mn^2^⁺ can act as dopants that enhance visible-light absorption, suggesting that controlled impurity retention during leaching may be beneficial rather than detrimental [83, 85]. This perspective contrasts with traditional pigment production, where ultra-high purity remains paramount.

Several underexplored areas are identified in the field of ilmenite processing for nanostructured TiO_2_ production: (1) Lifecycle assessment (LCA) of various ilmenite processing routes tailored specifically for nanostructured TiO_2_. (2) Development of continuous, intensified leaching reactors, such as those assisted by sonochemistry, microwaves, or pulsed electric fields. (3) Integration of electrolytic or photocatalytic steps for in-situ acid regeneration and metal recovery within the process flow. (4) Systematic studies that directly link leaching parameters to the photocatalytic performance of the resulting TiO_2_ nanostructures.

Considering resource availability and economic constraints, the sulfate process remains the most practical and accessible method for the extraction of TiO_2_ from low-grade ilmenite, striking a balance between cost, efficiency, and industrial applicability. Table 2 provides a systematic comparison of major titanium extraction and production routes, including the sulfate process, chloride process, Kroll process, FFC Cambridge process, and direct mechanochemical methods. Each route is evaluated in terms of feedstock suitability, key chemical mechanisms, operating conditions, final product characteristics, advantages, and disadvantages. For instance, the sulfate process is shown to be suitable for low- to medium-grade ilmenite and operates at 80–120 °C, whereas the chloride process requires high-grade feedstock and involves chlorination at 900–1000 °C. This comparative framework underscores the technical and economic trade-offs between different extraction strategies, particularly when targeting nanostructured TiO_2_ versus pigment-grade or metallic titanium.

**Table 2:** Flowchart of titanium metal and titanium dioxide pigments production process [45]

Process route	Feedstock	Main chemical mechanism	Key processing conditions	Final product	Advantages	Disadvantages	References
Hydrometallurgy (sulfate process)	Low- to medium-grade ilmenite (TiO_2_ 40–65%)	Acid leaching with H_2_SO₄, formation of TiOSO₄, hydrolysis, calcination	80–120 °C, concentrated H_2__2_SO₄, 1–3 h	Anatase TiO_2_	Suitable for low-cost feedstocks, well-established and widely used technology, lower initial investment	Generates large amounts of acidic waste, lower purity compared to chloride route, requires FeSO₄ removal	[22, 23, 31, 46]
Hydrometallurgy (chloride process)	Rutile, Ti slag, high-grade ilmenite	Chlorination in the presence of carbon to produce TiCl₄, followed by purification and oxidation	Chlorination at 900–1000 °C, Cl_2_ gas, coke, fluidized bed reactor	High-purity rutile TiO_2_	High-purity final product, suitable for sensitive industrial applications, less waste compared to sulfate route	High cost, requires high-grade feedstock, safety hazards (Cl_2_, TiCl₄)	[22, 23]
Thermo/Electrochemical (kroll process)	Natural or synthetic rutile with high purity	Reduction of TiCl₄ with Mg or Na under vacuum to produce Ti sponge	800–1000 °C, under vacuum or inert atmosphere	Titanium metal (Ti sponge)	The only industrial method for producing metallic titanium, suitable for strategic applications such as aerospace and medical	Energy-intensive, costly, batch process with long reaction times	[22, 23]
Electrometallurgy (FFC Cambridge Process)	Pure TiO_2_ or porous feedstock suitable for electrolysis	Direct electrolytic reduction of TiO_2_ at a cathode in molten CaCl_2_	~ 900 °C, electrolytic cell with graphite or titanium electrodes	Titanium metal	High efficiency, eliminates TiCl₄ and reduces the number of processing steps	Currently at lab or pilot scale, requires expensive electrolytes and specialized equipment	[22, 23]
Direct Mechanochemical Route	Natural ilmenite	High-energy mechanical milling increases surface area and alters structure, enhancing leachability	High-energy ball milling (30–600 min), adjustable milling speed and ball-to-powder ratio	Activated (pre-treated) ilmenite	Improves dissolution efficiency in subsequent leaching and reduces chemical consumption	Requires significant mechanical energy, potential contamination from milling media	[47, 48]

A detailed comparative analysis of various ilmenite leaching routes, including acid types, concentrations, temperatures, solid/liquid ratios, and special conditions, is compiled in Table 3. This table reveals several critical trends: (1) Reductive leaching with HCl and Fe⁰ achieves near-complete dissolution of iron and titanium, yielding a TiO_2_-rich residue suitable for nanotube synthesis [49]. (2) Acid mixtures (e.g., HF + H_2_SO₄) enhance extraction from refractory ores but may introduce fluoride ions that affect subsequent crystallization [56]. (3) Post-leaching treatments such as hydrolysis in alcoholic media favor anatase formation, while aqueous hydrolysis with high-temperature calcination promotes rutile [73]. The data in Table 3 underscore the strong influence of leaching parameters on both extraction yield and final TiO_2_ phase, providing a valuable reference for tailoring processes toward specific photocatalytic or pigment applications. While acid concentration, temperature, and solid–liquid ratio were consistently identified as the most influential parameters, the incorporation of additives, reductants, and post-treatment steps (e.g., hydrolysis or calcination) also played a crucial role in enhancing product quality and selectivity. Notably, the formation of anatase, rutile, or mixed phases is strongly correlated with hydrothermal conditions and solvent environments. This consolidated table provides a comprehensive benchmark for evaluating process efficiency and supports future optimization strategies for tailoring TiO_2_ phases from ilmenite feedstocks in industrial and environmental applications.

**Table 3:** A comparative analysis of various ilmenite processing routes, leaching conditions, and the resulting TiO_2_ phases is presented

Acid/Solution	Concentration	Temperature (°C)	Time	S/L	Special conditions / Process notes	Outcome (yield/purity)	Final TiO_2_ phase	Refs.
HCl	12 M	80	2.5 h (→ 1.5 h with Fe⁰)	1/20 (→ 1/8 with Fe⁰)	− 200 mesh; reductive leach with 0.1 kg Fe⁰ per kg ilmenite	Near-complete dissolution of Ti & Fe	–	[49]
HCl; H_2_SO₄	HCl 6 M; H_2_SO₄ (conc.)	100; 120	8 h; 0.5 h	1 g:100 mL; 0.5 g:20 mL	Sonochemical assistance; calcination at 950 °C	Synthetic rutile > 93% (HCl) and > 91% (H_2_SO₄); particle size 83–85 nm	Rutile (nanoparticles)	[50]
HCl	20 wt% HCl	25, 84, and 105	6 h	1/50	Preoxidation + Fe⁰	Selective Fe removal	Crystalline rutile	[51]
H_2_SO₄; HCl	8% (v/v)	95	2 h	¼	Titanium slag (− 100 μm) leached under identical S/L and time	TiO_2_ concentrate: 86.8% (H_2_SO₄) vs 91.0% (HCl); Fe_2_O_3_ reduced to 1.87% vs 0.61%	–	[52]
HCl	4–8 M	105	2 h (after 20 min boiling)	— (Fe⁰ 1 g per 5 g ore)	Pre-oxidation at 900 °C; reductant Fe⁰; hydrolysis in 2-propanol:H_2_O = 8:2	Ilmenite dissolution up to 86% @ 8 M; TiO_2_ gel yield ~ 32.7%	Anatase	[53]
H_2_SO₄	8–12 M	200	2 h	1:1	Two-stage leaching after NaOH mixing; roasting at 400 °C	TiO_2_ in residue: 32.3% rutile + 11.5% anatase (at 12 M)	Mixed (rutile + anatase)	[54]
H_2_SO₄	2.4 M (opt.)	85	≈3.9 h (235 min)	L/S = 15	RSM/CCD optimization; stirring 250 rpm; d80 ≈ 106 μm	Maximum TiO_2_ extraction 81.32%	–	[55]
HCl	-	105	5 h	1:1	MA + HCl leach + calcine	High-SSA nanorods; durable	–	[56]
H_2_SO₄ (leach) + D2EHPA (LLE)	H_2_SO₄ 45% v/v; D2EHPA 5–15% v/v	130 (leach); 35 (LLE)	4 h; 20 min	Ore/acid 10% w/v; O:A = 1:1	Autoclave leach; kerosene diluent; NH₄F stripping; calcination at 900 °C	Ti extraction ~ 99%; Fe uptake ~ 3.8% (lowest at lowest D2EHPA)	Anatase (after calcination)	[57]
HCl (reductive leach)	5–8 M (opt. 6 M)	80	6 h	1/20; 44–77 μm	Pre-oxidation 700 °C; Taguchi design; minimize Ti co-dissolution	Fe extraction 98.07%; Ti co-extraction 11.35%; residue 92.6% rutile	Rutile (synthetic grade)	[58]
HCl (× 5) → H_2_SO₄ (4 M) → NH_3_	HCl 37% (five digestions); H_2_SO₄ 4 M	90 (HCl); 60 (H_2_SO₄)	5 h + 12 h	—	pH adjustment to 10 (NH_3_); anneal 600/800 °C; photocatalysis tests	Active photocatalysts under LED/sunlight	Composite: TiO_2_ + Fe_2_TiO₅ (± α‑Fe_2_O_3_)	[59]
H_3_PO₄ → NH_3_	H_3_PO₄ 85%; NH_3_ 25%	Reflux; 500–900 (calc.)	5 h (digest)	—	TOP precipitation; ammonia washing; calcination	TiO_2_ ~ 88–90% (XRF); photocatalytic parity with commercial	Anatase @500 °C → Rutile > 800 °C	[60]
HCl (closed system) → NaOH + H_2_O_2_	HCl 6 M; NaOH 0.5 M; H_2_O_2_ 30%	170 (autoclave); 40 (reflux)	3 h; 1 h	–	Rotary autoclave; CTAB (HDTMA) templating; calcination 350/650 °C	100% TiO_2_ amorphous → > 99% anatase (350 °C) → ~ 100% rutile (650 °C)	Amorphous → Anatase → Rutile	[61]
H_2_SO₄	up to 78% (v/v)	150	6 h	–	Hydrolysis at 90 °C; calcination 1000 °C for 2 h	Product grade ~ 77.75% TiO_2_	Rutile (post‑calcination)	[62]
HCl	HCl pure	–	2 h	–	Fractional factorial DOE + pre-oxidation (900 °C, 120 min) + AAS	Optimization; pre-oxidation detrimental	–	[63]
HCl	12 M (reported; some text 2 M)	70–110	2 h	1:4	NaHCO_3_ pre‑mix; roasting 700 °C; water leach (50 mL)	Max TiO_2_ ~ 60.7–61% @110 °C (XRF)	Rutile (dominant, per XRD trend)	[64]
HF → K_2_TiF₆ → NH_3_; then H_2_SO₄ (0.7 M)	HF 20%; NH_3_ 4 M; H_2_SO₄ 0.7 M	RT/80; 170 (24 h)	5 h (HF); 24 h (hydrothermal)	–	Hydrolysis to Ti(OH)₄; anneal 550 °C; sulfate functionalization	Nanoparticles ~ 22 nm; enhanced RhB photodegradation	Anatase (SO₄^2^⁻-modified TiO_2_)	[65]
HCl → NaOH/NH_3_ + H_2_O_2_	NaOH 1 M or NH_3_ 12.5 wt% + H_2_O_2_	40 (alkaline); 100 (HT)	0.5 h (+ HT)	–	Surfactant-assisted (HDTMA) soft templating; mesoporosity control (H3/H4)	Mesoporous TiO_2_ nanorods (30–60 nm)	– (likely anatase)	[66]
H_2_SO₄ (after oxidation)	(1 + 1) diluted	≈120 (393 K)	12 h	–	Fluidized-bed oxidation (1173–1223 K; 5% O_2_/Ar; 40 min); leach dissolves Fe_2_TiO₅	Rutile yield 84.2 mol%	Rutile	[67]
HCl (Fe removal) → NaOH + H_2_O_2__2__2__2_; template	HCl 6 M; NaOH 1 M + H_2_O_2_	100 (HCl); 120 (HT)	2 h; 16 h (HT)	–	HDTMA templating; EtOH/H_2_O; calcination 400 °C	Well‑ordered mesoporous TiO_2_; superior TC photodegradation	– (mesoporous anatase)	[68]
H_2_SO₄ (two‑stage counter‑current)	11 → 13 M	120 → 200	30 min + 30 min	L/S = 1/3 + 1/3 g·cm⁻^3^	Refractory anatase ore; 200 rpm; diffusion‑controlled kinetics	> 99% TiO_2_, 99% Al_2_O_3_, 97% Sc_2_O_3_ (leach); quartz retained in residue	–	[69]
HCl (with additives)	— (pH‑controlled)	Atmospheric; pre‑oxidation at 817 °C	–	–	Additives: MgSO₄ (catalyst), Fe⁰ (reductant), NH₄HF₄ (silica attack)	Leaching efficiency ↑ ~ 20%; impurity reduction ~ 80%	Pseudorutile → Rutile (residue)	[70]
HCl + Fe⁰ + MgSO₄	7.5 M (opt.)	90	1–2 h	1:10; 450 rpm; + 150 μm	Direct leach; additives enhance Fe dissolution and reduce Ti loss	Fe extraction 92.3% (1 h) → 98.6% (2 h); residue ~ 91.4% TiO_2_	Rutile	[71]
NH₄Cl (aeration leach after carbothermal reduction)	0.5 M	90	7 h	–	Carbothermal reduction at 1250 °C (Ar/CNG, 3 h); Becher‑type route	Fe dissolution 97%; residue ~ 81 wt% TiO_2_	Synthetic rutile (rutile)	[72]
H_2_SO₄ / HCl (filtrate → hydrolysis)	H_2_SO₄ 2–9 M; HCl 4–8 M; hydrolysis in HCl 0.1–1 M or 2‑propanol:H_2_O = 9:1	60 (hydrolysis); up to 550 (calc.)	–	–	Alcoholic medium favors anatase crystallites up to 550 °C; transforms > 550 °C	Phase-tunable TiO_2_ from leachates	Anatase (≤ 550 °C) → Rutile (> 550 °C)	[73]

The preceding sections detailed the pre-treatment and extraction of TiO_2_ from ilmenite, yielding TiO_2_-rich precursors suitable for nanostructure synthesis. The following sections explore how these precursors, derived from sulfate or chloride routes, can be engineered into multidimensional TiO_2_ nanostructures with tailored morphologies. The linkage between extraction purity, phase composition (anatase/rutile), and the resulting nanostructural properties is critical, as it directly influences photocatalytic performance and application suitability.

## Synthesis of multidimensional titanium dioxide (TiO_2_) nanostructures: a morphology-oriented framework

The morphology of TiO_2_ photocatalysts, spanning zero- to two-dimensional nanostructures, critically governs their photocatalytic efficiency. Variations in particle geometry and dimensionality significantly influence light-harvesting capability, charge carrier separation, and interfacial reaction kinetics. Attaining such structural versatility necessitates a broad array of synthetic approaches, wherein meticulous modulation of factors including precursor concentration, pH, reaction temperature, duration, and chemical additives enables precise control over particle size, shape, crystallinity, and composition [74–76]. Importantly, the nature of the TiO_2_ precursor, whether derived from ilmenite via sulfate or chloride routes, imposes distinct constraints on phase purity and impurity profiles, thereby influencing achievable morphologies and reproducibility. This section adopts a structure-centered framework, organizing the discussion around dimensionality (0D, 1D, 2D) and linking each morphology to its synthesis pathway, structural characteristics, and functional implications.

**Fig. 3:**
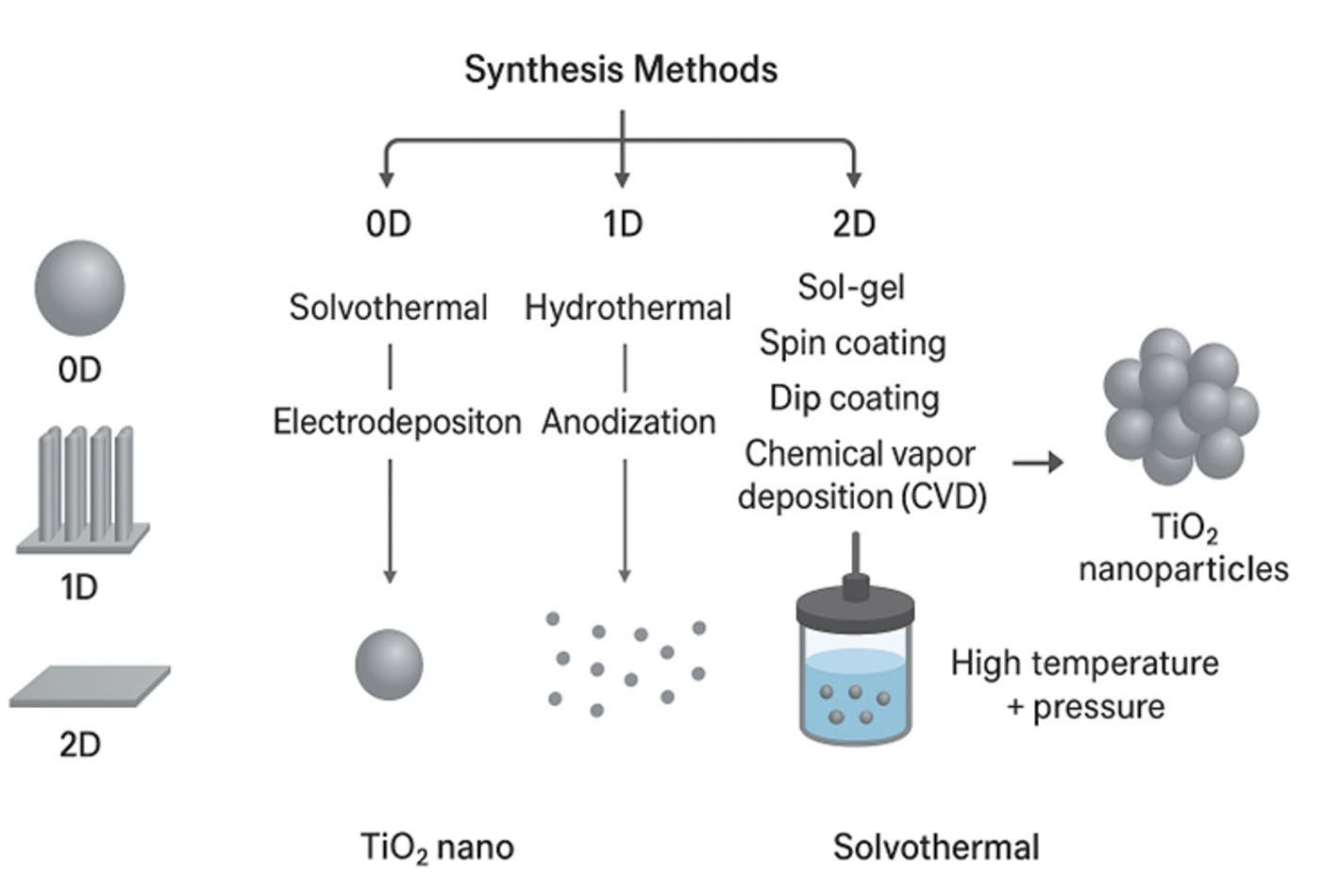
Schematic of various methods for synthesis of TiO_2_ nanoparticles with different sizes

In this section, we intentionally move beyond a generic “method-by-method” overview of TiO_2_ synthesis and adopt a structure-centered framework for multidimensional TiO_2_ architectures (0D/1D/2D). The discussion is therefore organized around structural-composition descriptors, including dominant phase (anatase/rutile/mixed; titanate-related phases where relevant), crystallinity and crystallite-size tendencies, defect chemistry (when reported), and textural parameters such as surface area/porosity and interface quality, because these parameters ultimately govern performance across application classes. This approach is consistent with the established understanding that morphology and microstructure, rather than synthesis label alone, determine photocatalytic effectiveness and functionality in different use scenarios [74–76]. Figure 3 schematically summarizes the various synthesis methods used to produce TiO_2_ nanoparticles, highlighting the correlation between specific processing routes and the resulting particle dimensions. Importantly, we emphasize that these structures are not discussed in isolation: ilmenite-derived precursor constraints (stemming from upstream pretreatment and extraction chemistry) shape attainable phase/morphology outcomes and influence reproducibility and scalability, which is a key point often underdeveloped in conventional TiO_2_ synthesis reviews [74–76].

### Zero-dimensional (0D) TiO_2_ nanostructures

Zero-dimensional TiO_2_ nanostructures, such as spherical nanoparticles, are particularly suited for photocatalytic applications due to their high specific surface area. When derived from ilmenite, the presence of residual Fe^3^⁺ or other transition metals can influence crystallization behavior and dopant incorporation, affecting optical and catalytic properties. Among the various 0D synthesis approaches, solvothermal, hydrothermal, and electrodeposition techniques have proven most effective [74, 75].

From a structural-composition standpoint, 0D TiO_2_ nanostructures are typically discussed as “nanoparticles,” yet their functional behavior is governed by phase constitution and crystallinity (anatase-dominant vs. mixed anatase–rutile) together with crystallite size and surface/defect features. In solvothermal routes, the ability to tune nucleation and growth enables controlled crystallinity and size distribution, while the resulting product quality remains sensitive to precursor chemistry and post-treatment history [76–78]. Likewise, electrodeposition, although often introduced as a fabrication technique, must be interpreted structurally: the electrochemical environment can influence layer compactness, nanocrystallinity, and defect-mediated visible-light response in doped systems [80–82]. Therefore, in comparing 0D synthesis routes, meaningful assessment should explicitly interpret phase fraction, crystallinity/crystallite size, and surface structural features as the primary determinants of charge-carrier recombination and surface reaction kinetics [74–76].

#### Solvothermal synthesis

Solvothermal synthesis allows the controlled growth of single-crystal nanoparticles from non-aqueous solutions under elevated temperature and pressure in an autoclave. This method provides excellent control over particle size, crystallinity, and uniformity. For instance, titanium butoxide or additives such as NH₄HCO_3_ and linoleic acid have been used at 150 °C for 24 h to produce stable anatase TiO_2_ nanoparticles capable of redispersion in various solvents. Ramachandran et al*.* reported 5 nm anatase TiO_2_ nanoparticles with photocatalytic efficiencies exceeding 96% in degrading methyl orange and methylene blue. Moreover, this method supports phase transformations and thermal treatments; however, its limitations include high equipment cost, safety considerations, and the inability to perform in-situ monitoring [77, 78].

#### Electrodeposition

Electrodeposition has emerged as a versatile, cost-effective, and controllable approach for synthesizing TiO_2_ nanoparticles [79]. In this technique, an electric current applied between a working electrode and a counter electrode drives the deposition of metal ions from the electrolyte onto the substrate. Critical parameters such as current density, deposition time, electrolyte composition, stirring rate, and polarization mode (continuous or pulsed) dictate the uniformity, thickness, and morphology of the deposited layer [80, 81].

Recent studies have successfully demonstrated the electrodeposition of TiO_2_ nanoparticles onto multiwalled carbon nanotube arrays using an electrolyte composed of 10 mM H_2_O_2_, 3 M KCl, and 10 mM Ti(SO₄)_2_. Optimal deposition occurred at 0.1 V for 30 min, with precise control over temperature, pH, and reaction duration to fine-tune particle size, crystallinity, and morphology. Electrodeposition has also been exploited to incorporate metal nanoparticles onto TiO_2_ surfaces, enhancing visible-light-driven photocatalytic activity. For instance, Lou et al*.* deposited silver nanoparticles onto TiO_2_ nanotube arrays, where the plasmonic effect of silver significantly improved charge carrier migration, resulting in enhanced CO_2_ reduction under visible light compared to pristine TiO_2_ [82].

**Fig. 4:**
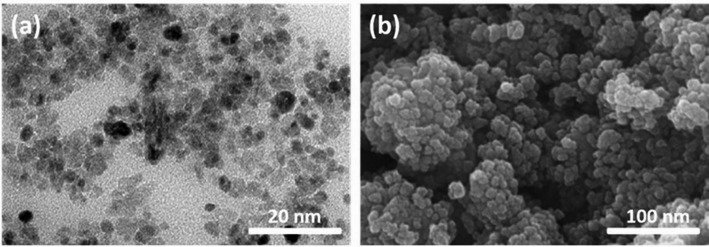
TEM (**a**) and SEM (**b**) images of zero-dimensional titanium dioxide (TiO_2_) nanoparticles. Reproduced with permission from [86]

Electrodeposition is particularly appealing due to its low operational temperature, relatively simple experimental setup, and scalability. However, it faces challenges including slow deposition rates and limited substrate compatibility. Hydrothermal synthesis has also been employed to produce TiO_2_ nanoparticles, often exhibiting superior visible-light photocatalytic performance relative to pristine TiO_2_, as demonstrated in Fe-TiO_2_ [83], Co/Cu-TiO_2_ [84], and S-TiO_2_ [85]. The resulting electrodeposited TiO_2_ nanoparticles have been characterized using SEM and TEM, revealing detailed nanoscale morphology, particle size distribution, and surface structural features, as presented in Fig. 4 [86].

### Synthesis methods of one-dimensional TiO_2_ nanostructures

One-dimensional (1D) TiO_2_ nanostructures including nanofibers, nanorods, nanotubes, and nanobelts represent a unique class of photocatalytic materials. Their elongated geometries, combined with high surface-to-volume ratios, enhance light absorption, charge separation, and surface reactivity compared to zero-dimensional counterparts. These characteristics make 1D TiO_2_ architectures highly attractive for applications such as environmental remediation, solar energy conversion, and photocatalytic hydrogen production. Among the commonly employed fabrication strategies, hydrothermal synthesis, electrochemical anodization, template-assisted growth, and sol–gel methods are particularly prominent.

One-dimensional TiO_2_ architectures (nanotubes, nanorods, nanofibers, and related morphologies) must be evaluated not only by their elongated geometry but also by their phase constitution and microstructural order, because these features dictate whether the 1D framework functions primarily as a high-surface-area scaffold or as a directed pathway for charge transport. Hydrothermal synthesis, for example, is widely employed because it promotes directional growth and enables systematic morphology control through precursor concentration, pH, temperature, and time; however, its outputs can vary significantly in crystallinity and phase stability depending on reaction and post-treatment conditions [87–91]. Microwave-assisted hydrothermal processing further modifies this structural outcome by accelerating kinetics and enabling rapid crystallization under relatively mild conditions, where phase formation (anatase in acidic media versus titanate-related phases in alkaline media) and crystallite-size trends have been explicitly reported [92–99]. Accordingly, the revised text in this section emphasizes that the structure–function coupling in 1D systems should be discussed through phase evolution (anatase ↔ rutile or titanate-related transformations), crystallinity/crystallite size, and architecture metrics (e.g., diameter/wall thickness for tubes), rather than by morphology names alone [87–99].

#### Hydrothermal method

Hydrothermal synthesis is widely recognized as an effective and versatile route for producing 1D TiO_2_ nanostructures [45, 87]. This method is particularly compatible with ilmenite-derived precursors, as the hydrothermal environment can accommodate residual impurities and promotes phase-pure anatase or rutile formation, depending on pH and temperature, factors previously discussed in the context of post-leaching treatments (Sect. 3.3).

A variety of 1D morphologies including nanotubes, nanorods, nanowires, and nanobelts can be obtained by carefully adjusting parameters such as precursor concentration, pH, temperature, and reaction time. Structural features, including tube length, diameter, and crystallographic orientation, play a decisive role in photocatalytic performance [88, 89]. For example, aligned nanotube arrays provide efficient pathways for charge carrier transport, minimizing recombination and improving the degradation of organic pollutants under UV or visible light.

**Fig. 5:**
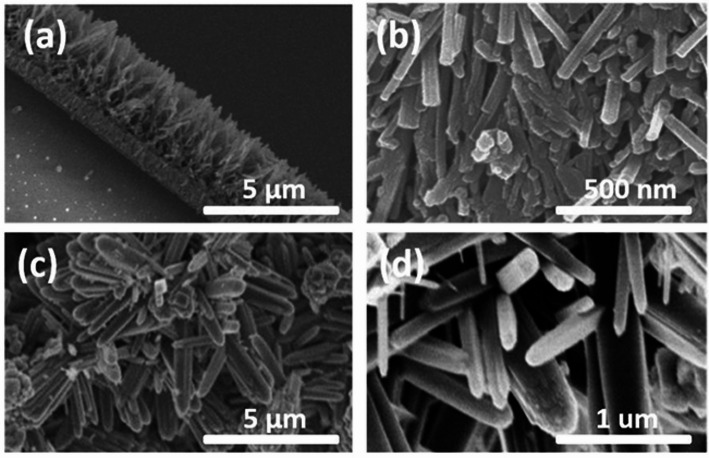
Different morphologies of one-dimensional (1D) TiO_2_ nanostructures synthesized by hydrothermal methods: **a**, **b** titanium dioxide nanotubes and **c**, **d** titanium dioxide nanorods. Reproduced with permission from [88, 91]

The hydrothermal approach offers advantages such as low operational costs, scalability, and moderate energy requirements. However, long reaction durations and heterogeneous particle sizes can pose challenges. To address these issues, hybrid strategies, combining hydrothermal treatment with subsequent annealing or solvothermal processing, have been applied, allowing controlled phase transitions (anatase to rutile) and precise tuning of nanostructure morphology, which ultimately enhances crystallinity and photocatalytic activity [88, 90]. Representative SEM and TEM images highlighting the structural diversity of hydrothermally synthesized 1D TiO_2__2_ nanostructures are presented in Fig. 5 [88, 91].

#### Microwave-assisted hydrothermal method

In recent years, microwave irradiation has emerged as a powerful tool to enhance hydrothermal processes, offering a more efficient route for the synthesis of nanostructured materials. Its ability to deliver rapid, uniform, and selective heating in dielectric media enables precise control over crystallographic phase evolution and morphological features of TiO_2_ [92–94].

**Fig. 6:**
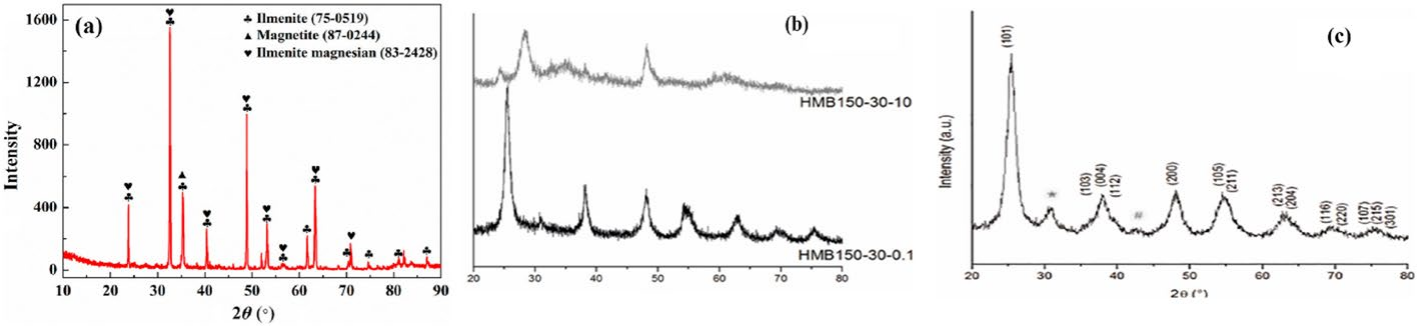
XRD patterns of **a** ilmenite, XRD patterns of TiO_2_ nanostructures synthesized under different chemical conditions: **b** samples prepared in alkaline medium showing the formation of titanate phases, and **c** samples synthesized in acidic medium indicating predominant anatase crystallization. Reproduced with permission from [99]

X-ray diffraction (XRD) for ilmenite is shown in Fig. 6a, all major diffraction peaks correspond to the reference pattern for ilmenite, confirming its trigonal crystal structure. TiO_2_ nanostructures were synthesized under both acidic and alkaline conditions using a microwave-assisted hydrothermal approach. In the acidic system, titanium tetraisopropoxide (TTIP) was introduced into HNO_3_ solution and subsequently processed in a Teflon-lined autoclave placed inside a microwave reactor, operating at 110–150 °C for 2–60 min. Under alkaline conditions, TTIP was treated in NaOH solutions of varying concentrations following a similar protocol [95, 96]. After synthesis, the resulting products were thoroughly washed, centrifuged, and dried.

**Fig. 7:**
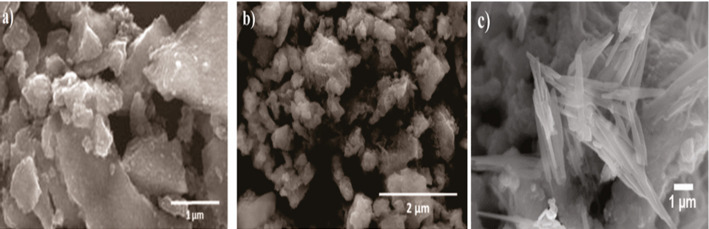
SEM images of TiO_2_ nanostructures: **a**, **b** samples synthesized under acidic conditions showing aggregated particles; **c** needle-like structures obtained under alkaline conditions. Reproduced with permission from [99]

As shown in Fig. 6b and c, X-ray diffraction (XRD) analysis confirms that the chemical environment critically dictates the crystallographic phase of the synthesized nanostructures. In acidic media, the products predominantly crystallize in the anatase phase with crystallite sizes of 5–6 nm, while under highly alkaline conditions, the phase composition shifts towards sodium titanates (Na_2_Ti₆O₁_3_ and Na_2_Ti_3_O₇). Correspondingly, the SEM images in Fig. 7 illustrate the morphological impact of this phase control: samples from acidic media show aggregated particles alongside amorphous Ti(OH)n domains, whereas those from alkaline conditions transform into distinct needle-like titanate nanotube structures. Together, these figures demonstrate the deterministic role of synthesis pH in governing both the crystalline phase and the final nanoarchitecture [97, 98].

This microwave-assisted hydrothermal route demonstrates significant advantages, including shorter reaction times, reduced energy consumption, and enhanced environmental compatibility. More importantly, the ability to tailor crystal phase and morphology provides a versatile platform for engineering TiO_2_ nanostructures with large surface area and tunable properties, making them promising candidates for photocatalysis, photovoltaic devices, and clean-energy technologies.

### Synthesis methods of two-dimensional TiO_2_ nanostructures

Two-dimensional TiO_2_ structures (thin films and coatings) must be discussed primarily through film microstructure, because 2D performance is governed by parameters that can be secondary in powder systems. Structural composition in films includes phase constitution (anatase/rutile/mixed), crystallinity and grain size, porosity/compactness, surface roughness, thickness uniformity, and interface quality with the substrate, features that determine optical behavior, adhesion/mechanical integrity, and charge-transport resistance. Spin-coating is widely adopted because it can produce uniform coatings rapidly, while film properties remain highly sensitive to parameters such as rotation speed, solvent/solute concentration, and post-treatment conditions [100–102]. Dip-coating similarly enables scalable, homogeneous films on complex substrates, and the final microstructure (crystallinity, porosity, roughness) and thickness tunability are explicitly linked to withdrawal rate, precursor concentration, and solution viscosity, although instabilities during withdrawal can cause defects and degrade film quality [103–105]. In addition, CVD offers strong control over film architecture and has been used to engineer doped structures and performance-relevant morphologies, reinforcing that deposition conditions and post-treatment must be interpreted in terms of structural outcomes rather than technique labels [108–111]. For these reasons, Sects. 4 and 3 has been strengthened to explicitly connect processing parameters to microstructural descriptors and then to application-relevant performance [100–105, 108–111].

#### Spin-coating technique

Spin-coating is among the most widely employed and cost-effective techniques for fabricating thin films on various substrates, with broad applications in advanced technologies and industrial processes. Its popularity stems from the ability to rapidly produce uniform films with thicknesses ranging from a few nanometers to several micrometers. In this process, a droplet of precursor solution is dispensed onto the center of a substrate, which is then rotated at high speed. The centrifugal force spreads the solution evenly across the surface, and in some cases, additional precursor can be applied during spinning to further refine the film uniformity [100].

Film properties are highly sensitive to experimental parameters, including spin speed, viscosity of the solution, spinning duration, molecular weight, and solute concentration, all of which directly influence the thickness and morphology of the final layer. SEM and TEM investigations of TiO_2_ thin films synthesized via spin-coating consistently reveal smooth and uniform structural features. For instance, Pan et al*.* successfully fabricated highly ordered mesoporous cubic WO_3_/TiO_2_ thin films using this method. Remarkably, incorporation of 4 wt% WO_3_ significantly enhanced photocatalytic efficiency in the degradation of 2-propanol under light irradiation [101].

**Fig. 8:**
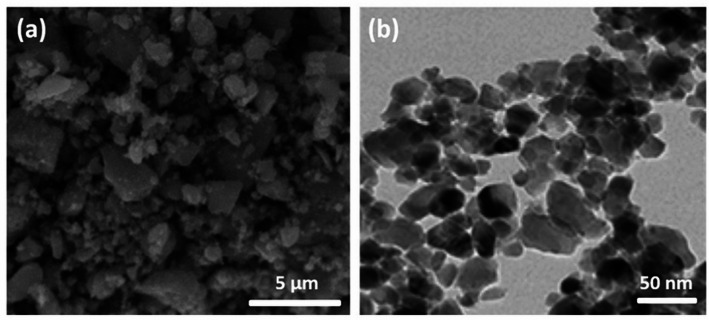
**a** SEM image and **b** TEM image of TiO_2_ thin film synthesized by spin coating method. Reproduced with permission from [102]

The technique offers notable advantages, particularly the ease of tuning film thickness by adjusting either spin speed or precursor viscosity. However, it also presents limitations: large substrates are difficult to process due to challenges in maintaining high rotational speeds, and the material utilization efficiency is relatively low, with only about 2–5% of the precursor adhering to the substrate while the remainder is discarded [101]. Representative SEM and TEM micrographs of spin-coated TiO_2_ thin films are provided in Fig. 8, illustrating their structural uniformity and nanoscale features [102].

#### Dip-coating method

Dip-coating is a cost-effective approach, particularly valued for its scalability and ability to produce homogeneous coatings over substrates of diverse geometries [103]. The technique relies on a straightforward sequence: immersion of the substrate into a precursor solution, controlled retention for a specific period, and subsequent withdrawal at a regulated speed. During this process, a uniform liquid layer adheres to the surface, which upon drying and post-treatment yields the desired thin film [104]. The ultimate microstructure, encompassing parameters such as crystallinity, porosity, and surface roughness, can be carefully tailored, while the coating thickness is highly tunable through modulation of withdrawal rate, precursor concentration, and solution viscosity [105]. Nevertheless, even minor instabilities, including vibration or mechanical disturbances during immersion and withdrawal, may induce streaks or surface irregularities, thereby compromising film quality.

**Fig. 9:**
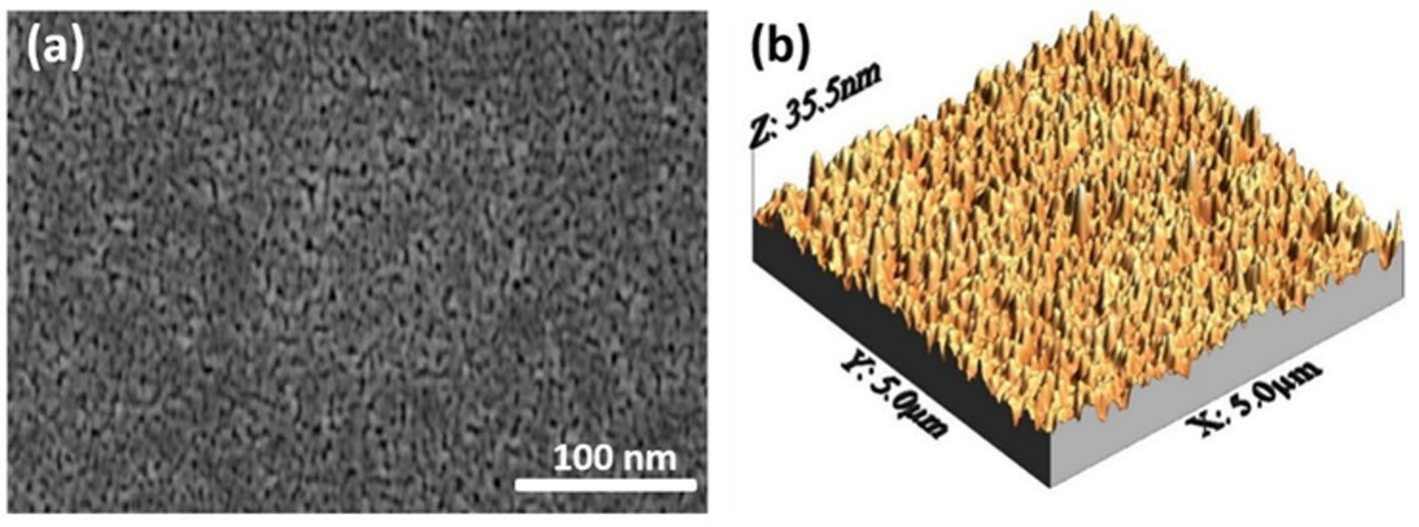
**a** SEM Image of TiO_2_ thin film synthesized by dip coating method, and **b** AFM images of TiO_2_ thin film. Reproduced with permission from [107]

A substantial body of literature demonstrates the applicability of dip-coating for the synthesis of TiO_2_-based thin films and nanocomposite architectures. For example, copper incorporation into TiO_2_ matrices has been reported to markedly improve visible-light-driven photocatalytic performance, with an optimum Cu/Ti ratio of 0.017 delivering the highest activity. Such investigations highlight the capability of dip-coating to facilitate compositional tuning while preserving structural uniformity. Despite its intrinsic simplicity and compatibility with sol–gel chemistry, however, challenges remain, particularly in achieving dense, defect-free coatings under certain processing regimes [106]. Surface characterization studies, including scanning electron microscopy (SEM) and atomic force microscopy (AFM), further confirm the nanoscale uniformity and morphological consistency of TiO_2_ thin films prepared through dip-coating Fig. 9 [107].

#### Chemical vapor deposition (CVD) method

Chemical vapor deposition (CVD) is recognized as a powerful and versatile technique for the fabrication of TiO_2_ thin films, offering a balance of cost efficiency, scalability, and structural precision. In this process, volatile precursors in the gas phase undergo controlled chemical reactions or thermal decomposition, leading to the deposition of solid layers on the substrate surface. The method is particularly valued for its ability to produce conformal coatings with adjustable thickness and microstructure under relatively mild operating conditions, making it attractive for both research and industrial applications [108].

Morphological analyses have highlighted the high tunability of CVD. For example, SEM observations revealed that a deposition time of one minute produced spherical TiO_2_ nanoparticles averaging ~ 20 nm in size with a film thickness of ~ 253 nm. In contrast, extending the deposition to two minutes resulted in markedly different star-shaped particles of ~ 200 nm and an increased film thickness of ~ 744 nm [109]. Such results demonstrate how deposition time alone can serve as a critical lever for tailoring particle morphology and film architecture.

Beyond simple film growth, CVD has been utilized to engineer advanced TiO_2_ structures. Mesoporous TiO_2_ films synthesized from titanium isopropoxide and water vapor precursors exhibited enhanced thermal stability without compromising chemical integrity [110]. Moreover, plasma-enhanced CVD (PECVD) has enabled the development of nitrogen-doped anatase films with preferred crystallographic orientation, using titanium isopropoxide as the titanium source and NH_3_ as the nitrogen donor. These N-doped films displayed superior photocatalytic efficiency in the visible-light-driven degradation of stearic acid [111].

**Fig. 10:**
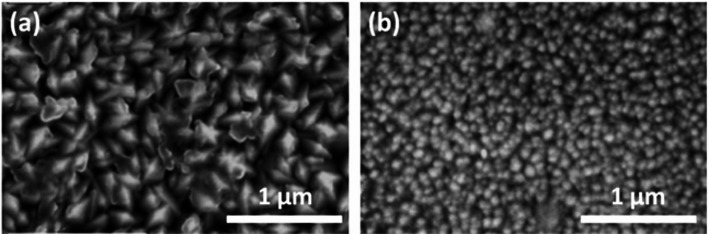
Scanning electron microscope (SEM) images of TiO_2_ at different deposition times: **a** one minute and **b** two minutes. Reproduced with permission from [109]

While conventional CVD offers remarkable control over film quality, challenges remain in meeting the demands of next-generation nanofabrication, particularly in achieving three-dimensional uniformity and nanoscale precision. Hybrid approaches such as PECVD provide promising solutions to overcome these constraints. Representative SEM images of TiO_2_ thin films obtained at different deposition times are shown in Fig. 10 [109], illustrating the pronounced influence of process duration on particle morphology and film evolution.

## Advanced synthesis techniques for TiO_2_ nanostructures

The fabrication pathway for TiO_2_ nanomaterials directly governs their physicochemical characteristics, which in turn dictate performance in target applications. Synthesis strategies can be broadly categorized by their approach, bottom-up (building from molecular precursors) versus top-down (breaking down bulk material), or by their processing medium, such as liquid-phase (e.g., sol–gel, hydrothermal) and vapor-phase methods. The choice of technique involves a balance between scalability, cost, and the precise control required over parameters like particle size, morphology, crystallographic phase, and surface chemistry. The following sections detail the most prominent synthesis methods employed for nanostructured TiO_2_, beginning with the highly versatile and widely researched sol–gel process.

### Electrochemical anodization

Electrochemical anodization represents an effective and economical approach for the fabrication of well-ordered TiO_2_ nanotubes and nanorods. This technique commonly employs a three-electrode configuration in an aqueous electrolyte. For example, TiO_2_ nanotubes have been successfully synthesized in an ethylene glycol-based solution under an applied voltage of 30 V for 6 h. Subsequent loading of silver nanoparticles via electrodeposition, followed by annealing at 550 °C, considerably enhanced their photocatalytic activity in CO_2_ reduction under visible light [82, 112].

In other studies, self-assembled TiO_2_ nanotube arrays were produced using a two-electrode system, with titanium serving as the anode and stainless steel as the cathode. The electrode spacing was maintained at 2 cm, while the anodization potential and annealing temperature were systematically varied to examine their effects on nanotube diameter and crystallographic orientation. Results indicated that these two parameters are pivotal in controlling both the dimensions and crystal structure of the nanotubes. The fabricated arrays demonstrated stable photocatalytic behavior, effectively decomposing metoprolol (a β-blocker) under UV-LED irradiation. Although this method is cost-effective and enables the formation of high surface-area nanotubes, its limitations include long processing times and challenges associated with scaling up production [113].

### Sol–gel method

The sol–gel technique is widely recognized as a simple yet versatile approach for producing one-dimensional TiO_2_ nanostructures, such as nanorods, nanotubes, and nanowires. This low-temperature process is based on the hydrolysis of titanium alkoxides followed by condensation, forming a colloidal sol that eventually transforms into a gel upon thermal treatment. The addition of acidic (HCl) or basic (NaOH) catalysts can accelerate the reaction kinetics. By adjusting key parameters including pH, solvent type, precursor selection, and catalyst concentration, it is possible to precisely control the size, morphology, and crystalline phase of TiO_2_, yielding nanostructures with high purity and uniformity [114, 115].

Recent work has demonstrated the synthesis of N-doped TiO_2_ nanorods using an aqueous sol–gel method combined with hydrothermal treatment. Titanium tetraisopropoxide, diethanolamine, and H_2_O_2_ served as precursors, while trifluoroacetic acid (TFA) was introduced as a growth modulator. FESEM analysis revealed that incorporating 1% TFA promotes uniform nanorod formation by acting as both a pH regulator and a selective inducer of the rutile phase. The resulting N-doped TiO_2_ nanorods displayed outstanding photocatalytic performance in methylene orange degradation and solar-driven hydrogen evolution.

**Fig. 11:**
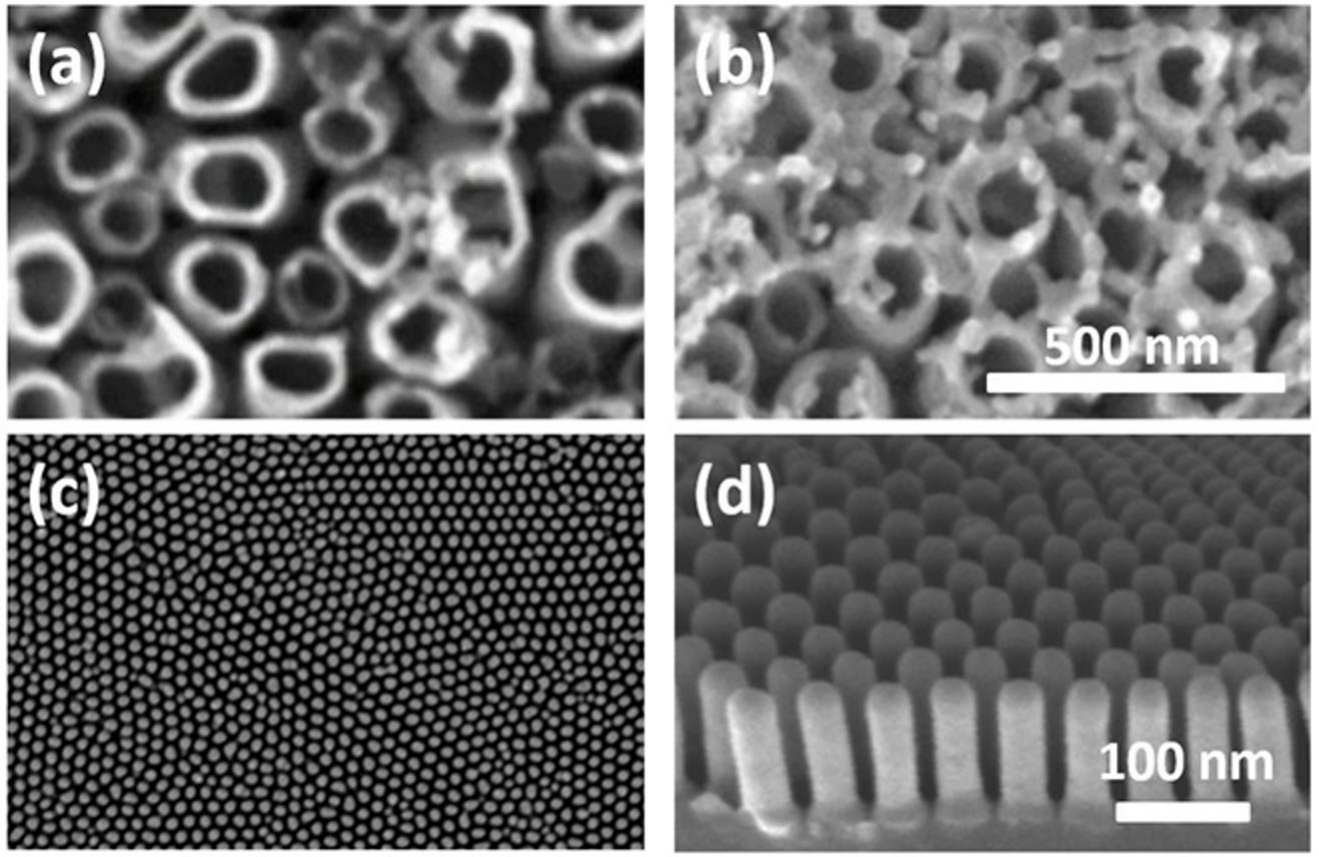
FESEM images of **a**–**b** top view of Ag/TiO_2_ nanotube arrays synthesized by electrochemical anodizing method. Reproduced with permission from [56]. **c**–**d** top view and cross-section of TiO_2_ nanotube arrays prepared using the template-assisted method. Reproduced with permission from [117]

The sol–gel route is valued for its versatility, enabling fine control over porosity, particle size, and molecular-level mixing, while consuming less energy compared to other methods. However, limitations such as high precursor costs and the time-consuming nature of gelation present challenges for large-scale implementation [116]. Representative FESEM images of one-dimensional TiO_2_ nanostructures are presented in Fig. 11, showing (a–b) top-view of Ag/TiO_2_ nanotube arrays synthesized via electrochemical anodization [82] and (c–d) top-view and cross-sectional views of TiO_2_ nanotube arrays prepared using the template-assisted method [117].

The synthesis methods reviewed herein demonstrate the feasibility of producing tailored TiO_2_ nanostructures from ilmenite-derived precursors. However, transitioning from laboratory-scale synthesis to industrial application reveals several persistent challenges, including scalability, reproducibility, and energy efficiency. The following section identifies key research gaps and proposes future directions to bridge these divides, emphasizing the need for integrated process design that connects feedstock characteristics, extraction efficiency, and nanostructure functionality.

## Challenges and future directions: bridging lab to industry

The extensive adoption of titanium dioxide across multiple industries is driven by a unique confluence of properties, including high chemical and photostability, non-toxicity, potent photocatalytic activity under UV illumination, and favorable electronic band structure. Engineering TiO_2_ at the nanoscale amplifies these inherent traits and can unlock new functionalities through increased surface area, quantum confinement effects, and tailored morphologies. This section reviews the principal application domains where nanostructured TiO_2_ has made a transformative impact, commencing with its most prominent role in photocatalytic environmental remediation.

The extrapolation of ilmenite-derived titanium dioxide (TiO_2_) nanostructures from a laboratory novelty to an industrial commodity encounters a spectrum of formidable challenges, necessitating a paradigm shift in both processing philosophy and engineering implementation. Primarily, the intrinsic compositional heterogeneity of natural ilmenite deposits constitutes a formidable impediment to process reproducibility. Unlike synthetic precursors, natural ilmenite exhibits a profound variability in elemental constitution, fluctuating in Fe, Mn, Mg, and Si content, depending on the geographical provenance of the ore [28]. This stochastic variability demands the development of flexible, adaptive processing flows and robust pre-treatment protocols [31] capable of accommodating such fluctuations without compromising the yield, phase purity [73], or photocatalytic efficacy of the final TiO_2_ product. Consequently, future research must prioritize the standardization of mechanochemical activation [36, 37] and hydrometallurgical strategies that can effectively homogenize these raw materials.

Concomitantly, the thermodynamic intensity and environmental footprint associated with conventional extractive metallurgy remain critical bottlenecks. While the established sulfate and chloride routes are operationally mature, they are often characterized by high energy inputs and the generation of substantial waste streams, such as FeSO₄ in the sulfate route or hazardous chlorine byproducts in the chloride route [22, 23]. To harmonize industrial production with global sustainability goals, there is an exigent need to pioneer "green" hydrometallurgical alternatives. Promising avenues include the exploration of low-temperature leaching (< 120 °C) utilizing organic acids [60], the intensification of mechanochemical activation [34], and the adoption of emerging techniques such as microwave-assisted leaching [56] and advanced solvent extraction methodologies [57] to enhance selectivity and reduce waste.

Furthermore, bridging the chasm between batch-oriented laboratory syntheses and continuous industrial manufacturing necessitates rigorous reactor engineering. While hydrothermal and solvothermal methodologies afford exquisite control over morphology at the bench scale, their translation to continuous throughput is fraught with complexities related to mass transfer limitations and the capital cost of high-pressure equipment [74, 75]. Future endeavors must focus on the design of continuous flow reactors and scalable wet-chemical regimes that preserve nanostructural integrity—such as high surface area and crystallinity, while meeting industrial volumetric demands.

Ultimately, the integration of comprehensive by-product valorization strategies is indispensable for economic viability. Conceptualizing ilmenite processing through a circular economy lens, where iron-rich residues are transmuted into pigments, fertilizers, or energy storage materials [71, 72], and silica byproducts are repurposed for construction, can transform waste liabilities into revenue streams. This holistic, cradle-to-cradle approach not only mitigates environmental externalities but also substantively enhances the cost-competitiveness of ilmenite-based TiO_2_ technologies relative to synthetic analogues [45].

## Conclusion

This review has provided a comprehensive analysis of the strategic pathway from raw ilmenite ore to advanced titanium dioxide (TiO_2__2_) nanostructures. The investigation underscores that while ilmenite represents an abundant and economically viable source of titanium, efficient utilization requires rigorous pre-treatment strategies, specifically mechanical activation such as ball milling, to overcome intrinsic heterogeneity and enhance reactivity. Comparative analysis of extractive routes reveals that while the sulfate process remains the most practical method for low-grade ores, the chloride process is superior for high-purity applications. However, emerging mechanochemical and hydrometallurgical routes offer promising, energy-efficient alternatives that could mitigate the environmental burdens associated with traditional large-scale acid leaching.

A core finding of this study is the critical relationship between synthesis methodology and nanostructure morphology. Adopting a morphology-oriented framework, we explored how zero-dimensional (0D), one-dimensional (1D), and two-dimensional (2D) architectures—fabricated via hydrothermal, microwave-assisted, solvothermal, and electrodeposition techniques—dictate the final photocatalytic performance. It is evident that achieving phase control (predominantly anatase for activity) and high surface area is essential for optimizing charge carrier dynamics. Consequently, the resulting nanostructures demonstrate significant efficacy in environmental remediation, particularly in the degradation of organic pollutants and photocatalytic water splitting under UV and visible light irradiation.

Despite these promising laboratory advancements, the transition to industrial scalability faces significant challenges. The variability in local ilmenite compositions and the high energy intensity of conventional processing remain persistent bottlenecks. Future efforts must therefore focus on developing continuous, low-energy processing flows and intensifying green chemistry principles, such as the valorization of iron byproducts. By bridging the gap between laboratory-scale nanostructure engineering and industrial process economics, ilmenite-derived TiO_2_ can fully realize its potential as a cornerstone of sustainable green technologies.

## Supplementary Information

Below is the link to the electronic supplementary material.

Supplementary Material 1.

## Data Availability

No datasets were generated or analysed during the current study.
